# The Long Coiled-Coil Protein NECC2 Is Associated to Caveolae and MODULATES NGF/TrkA Signaling IN PC12 CELLS

**DOI:** 10.1371/journal.pone.0073668

**Published:** 2013-09-06

**Authors:** Alberto Díaz-Ruiz, Yoana Rabanal-Ruiz, Andrés Trávez, Francisco Gracia-Navarro, David Cruz-García, Maité Montero-Hadjadje, Youssef Anouar, Stéphane Gasman, Nicolas Vitale, Rafael Vázquez-Martínez, María M. Malagón

**Affiliations:** 1 Department of Cell Biology, Physiology and Immunology, Instituto Maimónides de Investigación Biomédica (IMIBIC)/Reina Sofia University Hospital/University of Córdoba, Córdoba, Spain; 2 INSERM U982, Laboratory of Neuronal and Neuroendocrine Differentiation and Communication, Institute for Research and Innovation in Biomedicine, University of Rouen, Mont-Saint-Aignan, France; 3 Institut des Neurosciences Cellulaires et Intégratives (INCI), Centre National de la Recherche Scientifique (CNRS UPR 3212) and Université de Strasbourg, Strasbourg, France; 4 CIBER Fisiopatología de la Obesidad y Nutrición (CIBERobn), Spain; University of Santiago de Compostela School of Medicine - CIMUS, Spain

## Abstract

TrkA-mediated NGF signaling in PC12 cells has been shown to be compartimentalized in specialized microdomains of the plasma membrane, the caveolae, which are organized by scaffold proteins including the member of the caveolin family of proteins, caveolin-1. Here, we characterize the intracellular distribution as well as the biochemical and functional properties of the neuroendocrine long coiled-coil protein 2 (NECC2), a novel long coiled-coil protein selectively expressed in neuroendocrine tissues that contains a predicted caveolin-binding domain and displays structural characteristics of a scaffolding factor. NECC2 distributes in caveolae, wherein it colocalizes with the TrkA receptor, and behaves as a caveolae-associated protein in neuroendocrine PC12 cells. In addition, stimulation of PC12 cells with nerve growth factor (NGF) increased the expression and regulated the distribution of NECC2. Interestingly, knockdown as well as overexpression of NECC2 resulted in a reduction of NGF-induced phosphorylation of the TrkA downstream effector extracellular signal-regulated kinases 1 and 2 (ERK1/ERK2) but not of Akt. Altogether, our results identify NECC2 as a novel component of caveolae in PC12 cells and support the contribution of this protein in the maintenance of TrkA-mediated NGF signaling.

## Introduction

Neuroendocrine long coiled-coil protein 2, also referred to as Jakmip3, is the third and still uncharacterized member of a family of long coiled-coil, vertebrate-specific proteins composed of two additional members, Jakmip1/Marlin1 and NECC1/Jakmip2. Jakmip1/Marlin1 is predominantly expressed in neurons and lymphoid cells. In lymphocytes, Jakmip1 associates with microtubules and the non-receptor tyrosine kinases Tyk2 and Jak1 and has been proposed to participate in polarized secretion and segregation of signaling complexes [1]. Jakmip1/Marlin1 associates with microtubules and the molecular motor kinesin-1 [2,3] in neurons, wherein it has been suggested to play a role in the maintenance of the structural organization of the Golgi apparatus and in cell morphogenesis and migration [4]. Both *Necc1* and its ortholog, *Necc2*, are preferentially expressed in neuronal, neuroendocrine, and endocrine tissues [5]. Specifically, NECC1 locates to the Golgi apparatus in neuroendocrine PC12 cells and controls the regulated release of secretory cargo [6].

Long coiled-coil proteins represent highly versatile molecules that exert a wide variety of essential functions in eukaryotic cells [7]. They serve as tethering factors controlling the association of transport vesicles with target membranes as well as membrane pairing and fusion events. Long coiled-coil proteins have been also proposed to act as molecular scaffolds to maintain and organize intracellular compartments, including the nuclear envelope, the Golgi apparatus or the presynaptic active zone [7–11]. Given their ability to interact with multiple partners, these proteins have been suggested to integrate signals and transduction pathways [7]. Indeed, there is compelling evidence supporting the relevance of scaffolding factors in the organization of membrane microdomains specialized in transmembrane signalling, such as caveolae [12,13]. Caveolae are enriched in the integral membrane protein caveolin-1, and constitute localized platforms for downstream signaling of several tyrosine kinase receptors [14]. Specifically, the high affinity neurotrophin receptor TrkA, as well as many of the intermediates involved in the TrkA signaling cascade, have been shown to locate to caveolae or to caveolae-like membranes in PC12 cells and in TrkA-transfected NIH-3T3 cells [15,16]. Remarkably, the presence of long coiled-coil proteins in caveolae has not been documented yet.

Herein, we demonstrate that NECC2, whose expression and localization in PC12 cells is regulated by nerve growth factor (NGF), co-localizes with caveolin-1 and TrkA in the plasma membrane of these cells. Moreover, we show that both silencing and overexpression of *Necc2* impairs TrkA-dependent activation of extracellular-regulated kinases 1 and 2 (ERK1/ERK2) without modifying TrkA-dependent Akt phosphorylation. Taken together, our data indicate that NECC2 is a novel component of caveolae in PC12 cells, and support a role for this protein as a molecular scaffold modulating NGF-mediated TrkA signal transduction.

## Materials and Methods

### Antibodies and reagents

Two polyclonal antisera, anti-NECC2-I and anti-NECC2-II, were raised by rabbit immunization with synthetic peptides corresponding to amino acid residues 2-17 (SKKGAGSRAKGDKAE) and 434-447 (RYRKQRKKMAKLPK) of rat NECC2, respectively. Purified antibodies were obtained by immunoaffinity chromatography with the corresponding immobilized peptide. Monoclonal anti-cMyc antibody was purchased from Serotec (Oxford, UK). Monoclonal antibodies to GM130 and EEA1 were from BD Transduction Labs (Lexington, KY). Rabbit polyclonal anti-TrkA antibody was from Millipore (Billerica, MA) and mouse anti-γ-tubulin was from Thermo Fisher Scientific Inc. (Waltham, MA). Mouse anti-caveolin-1 was from Novus Biologicals (Cambridge, UK). Polyclonal antibodies to Akt, pAkt (Ser473), and p44/42 MAPK (T202/Y204) were from Cell Signaling Technology Inc. (Danvers, MA). Polyclonal antibody to ERK1/2 was from Santa Cruz Biotechnology (Heidelberg, Germany). Alexa Fluor-conjugated secondary antibodies, and Lipofectamine 2000 were from Invitrogen (Carlsbad, CA). NGF 7S was purchased from Chemicon (Temecula, CA). Latrunculin B was from Calbiochem (Darmstadt, Germany). Unless otherwise indicated, all other reagents were purchased from Sigma-Aldrich (Madrid, Spain).

### Computational analysis

Database search and prediction of alternatively spliced isoforms were performed using GNOMON (http://www.ncbi.nlm.nih.gov/projects/genome/guide/gnomon.shtml) and Ensembl Genome Browser (http://www.ensembl.org) websites. Analysis of amino acid sequences was carried out using ScanProsite, MotifScan, and Pfam algorithms included in the Expasy server (http://www.expasy.net/). Target regions for siRNA were designed using algorithms available on Promega website (http://www.promega.com/siRNADesigner/) and chosen according to the guidelines for effective siRNAs [17].

### Plasmid expression vectors and DNA constructs

Constructs encoding GFP-*Necc2* and cMyc-*Necc2* have been previously described [5]. To generate plasmid vectors containing truncated versions of NECC2, different fragments of mouse *Necc2* cDNA corresponding to amino acid residues 1-285 (Δ285), 1-372 (Δ372) or 1-825 (ΔCBD) fused to the C-terminus of cMyc were amplified by PCR and subcloned into pcDNA3 vector (Invitrogen). Untagged *Necc2* cDNA was also cloned into pcDNA3 vector. Plasmids coding for rat *TrkA* tagged with HA epitope and *Caveolin-1* were kindly provided by Dr. J.X. Comella (Vall d’Hebron Research Institute, Barcelona, Spain) and Dr. B. Chini (CNR Neuroscience Institute, Milan, Italy), respectively. *Caveolin-1* was subcloned in frame to the C-terminus of CFP in the pECFP-C1 vector.

A specific siRNA for silencing rat *Necc2* (5´-GTTTGTGCAGTTGCTTTAT -3´) was cloned using the BglII and HindIII sites in front of the H1- RNA promoter of the pEGFP-RNAi plasmid as described earlier [18].

### Cell culture and NGF treatment

PC12 cells derived from pheochromocytoma of rat adrenal medulla [ATCC (CRL-172)] and HEK-293 AD cells derived from human embryonic kidney 293 cells [ATCC (CRL-157)] were grown to 90% confluence and exposed for 4 h to low-serum differentiation media (DMEM containing 1% horse serum, 1% antibiotic/antimycotic solution, and 2 mmol/L of L-glutamine). Cells were then stimulated with NGF (0,76 mmol/L) for several time intervals, as indicated. For differentiation experiments, PC12 cells were seeded onto 25-mm poly L-lysine coated glass coverslips (25.000 cells/coverslip) and incubated in differentiation media containing 0,76 mmol/L of NGF for 4 days.

### Transfection and selection of stably shRNA-plasmid transfected PC12 cells

Transient transfection of cells was performed using Lipofectamine 2000. Transfected cells were used for experiments 24 h later. For short-term NGF treatments of cells overexpressing NECC2, T25 flasks of transiently transfected-PC12 cells were plated 1 day after transfection at a density of 100.000 cells onto 12-well plates. Cell viability was assessed by MTT assay according to the manufacturer’s instructions. To generate stably transfected cells, PC12 cells were transfected with 1 µg of pEGFP-RNAi or pEGFP-RNAi-*Necc2*. Twenty-four h later, transfected cells were selected with 1,4 µmol/L of geneticin. Selection efficiency was tested by flow cytometry for GFP expression, which was detected in 51% of the transfected cells

### RNA isolation and expression analysis by RT-PCR

Total RNA from PC12 cells was isolated using RNA-easy Mini Kit columns (Qiagen, Hilden, Germany), and a 2-µg aliquot of total RNA was reverse transcribed with the cDNA First-Strand Synthesis kit using random primers (Fermentas, Hanover, MD). The cDNA was used as a template for standard PCR amplification of rat *Necc2* using specific primers (forward 5´-CGGAAGAAGAGCGTGAGAAG-3´; reverse 5´-TCCCGTTAGAGGACAAAGGT-3´). Nested PCR was carried out to amplify alternative spliced *Necc2* isoforms using internal reverse primers (5´-GACCAGAGAATGAAAGCTAGGG-3´; 5´- TTCGCAGAGGGCTTTTCTTC-3´). Non-DNA controls were run to control for exogenous contamination. PCR products were purified (Bioneer Inc., CA), and sequenced (SCAI, University of Córdoba) to confirm identities of amplicons.

### Immunofluorescence and confocal microscopy

PC12 and HEK-293 AD cells grown on glass coverslips were processed for immunofluorescence and examined by confocal microscopy as described previously [6]. In a set of experiments, cells were exposed to 5 µmol/L of Latrunculin B (LatB) for 30 min and then processed for immunocytochemistry. Deconvolution of confocal images was carried out using the software package HUYGENS ESSENTIAL 2.4.4 (Scientific Volume Imaging, Hilversum, The Netherlands). Colocalization rates were determined by an overlapping pixel map of the two fluorescent channels (i.e., a mask) using the Colocalization Finder plugin for ImageJ 1.44 (NIH, Bethesda, MA) and Manders’ coefficient (MC) using Imaris software (Bitplane, Zurich, Switzerland).

### SDS-PAGE and immunoblotting

Whole-cell protein extracts were obtained by homogenization in RIPA buffer containing protease inhibitors. NGF-stimulated cells were lysed in SDS-dithiothreitol buffer [19]. 60 µg of total protein in RIPA buffer or 20 µl of SDS-DTT cell lysate were separated by SDS-PAGE and immunoblotted with specific antibodies. Densitometric analysis of the immunoreactive bands was carried out with ImageJ software. Quantitative data obtained with the anti-pAkt or anti-p42/44 MAPK sera were normalized against the corresponding total Akt or ERK values, respectively.

### Subcellular fractionation

Cytosolic (S) and crude membrane (P) fractions of PC12 cells were obtained by subcellular fractionation [6]. Protein distribution was analyzed by immunoblotting using 50 µg of protein of each fraction.

### Detergent-free Cell Membrane Fractionation

Caveolin-enriched membrane fractions were isolated from PC12 cells grown to confluency [15]. After ultracentrifugation, 9 fractions (450 µl) were collected from the top of the sucrose gradient and subsequently analyzed by SDS-PAGE.

### Statistical analysis

Data from PC12 cells were obtained from a minimum of three independent experiments. Statistical analysis was performed using GraphPad Prism 4 (GraphPad Software, Inc., La Jolla, CA, US). A one-way ANOVA followed by a statistical test for multiple comparisons (Newman-Keuls Multiple Comparison test) was applied to compare experimental treatments. For the analysis of the effects of NGF on TrkA signaling in the short-term, a Student’s t –test was used. Differences were considered statistically significant if p < 0.05. Data are expressed as mean ± S.E.M.

## Results

### Structural analysis of NECC2

**Figure 1:**
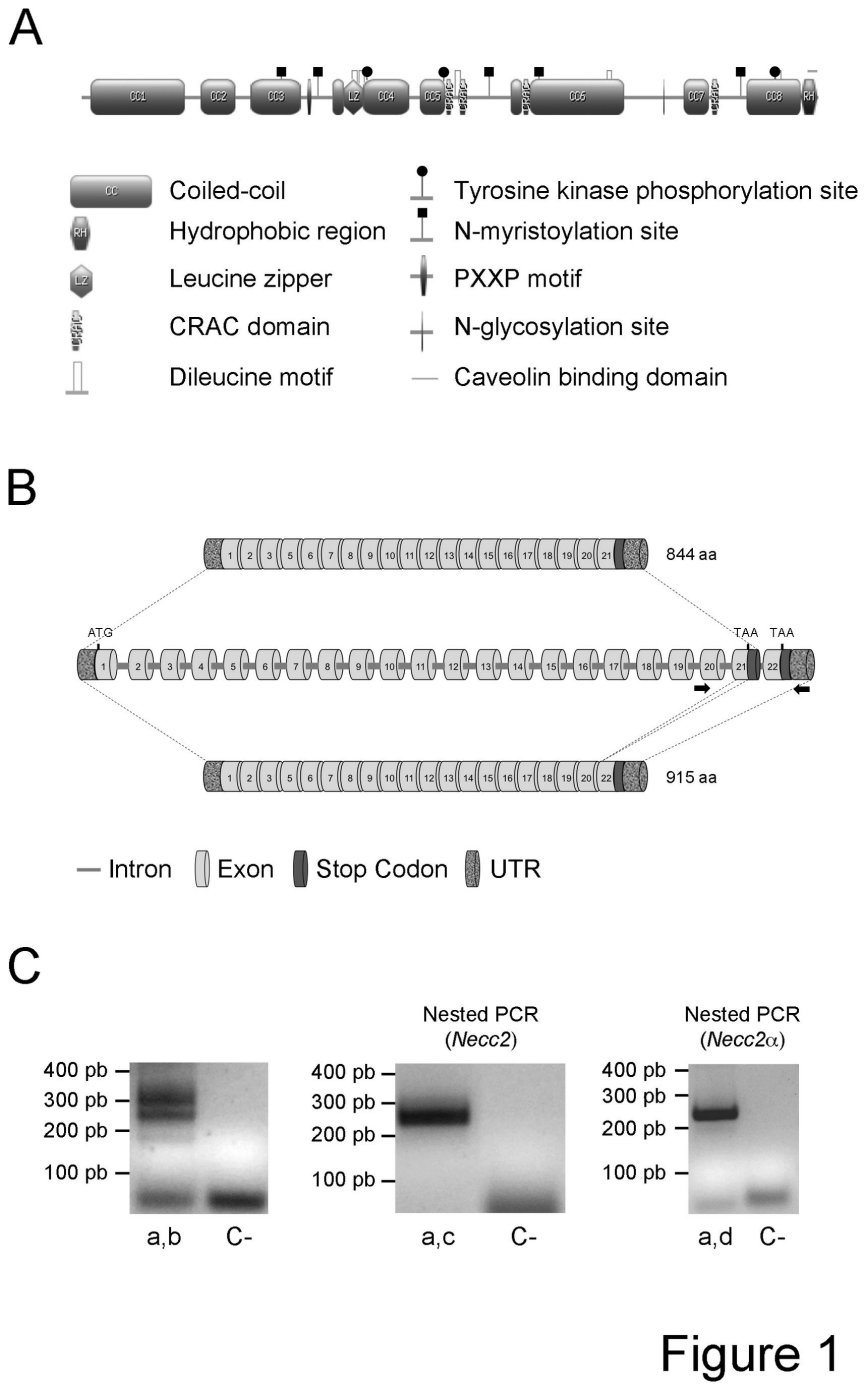
In silico analysis of rat NECC2 sequence. *A*. Schematic representation of the structural and functional motifs predicted in rat NECC2 amino acid sequence. *B*. Schematic representation of the genomic structure of rat *Necc2* coding for the *Necc2* isoform containing the HR domain and a newly identified transcript lacking this domain (*Necc2*α). Arrows indicate the location of the paired primers used to amplify both *Necc2* transcripts. *C*. Standard PCR amplification in PC12 cells shows two PCR products with the expected sizes for the two rat *Necc2* transcripts (left panel; primers a and b). Nested PCR amplification of *Necc2* transcript (central panel; primer c) or the *Necc2*α transcript (rightmost panel; primer d) using specific internal reverse primers. Non-DNA samples (C-) are shown as controls for exogenous contamination.

The general features of the NECC2 sequence have been already depicted [5]. Further characterization of the 19-amino acid hydrophobic region (HR) region revealed the presence of two overlapping caveolin-binding domains (CBD) [ØXØXXXXØ (amino acids 829 to 836), and ØXØXXXXØXXØ (amino acids 833 to 843), X being any amino acid, and Ø an aromatic amino acid (Trp, Phe, or Tyr)] [20] (Figure 1A).

NECC2 contains four predicted CRAC (cholesterol recognition/interaction amino acid consensus) domains [21], five dileucine motifs, which represent conventional signals for endocytosis [22], one leucine zipper domain, three potential tyrosine kinase phosphorylation sites, including one Akt- and two ERK-specific phosphorylation sites [23], one N-glycosylation site, and six N-myristoylation sites (Figure 1A). NECC2 also contains seven diacidic DXE (Asp-X-Glu) motifs, which mediate the export of proteins from the endoplasmic reticulum [24], and two PXXP domains, which act as target sequences for SH3-type domains [25].

Analysis of the genomic sequence of rat *Necc2* allowed us to identify a novel *Necc2* gene variant. This variant would originate by alternative intra-exonic splicing of exon 21, which codes for most of the C-terminal HR (residues 825–843). Alternatively spliced mRNAs would generate a short isoform of 844 amino acids containing the HR (98,7 kDa), and a 915 amino acids-isoform (106 kDa) containing an alternative C-terminus (Figure 1B), which were indeed amplified using specific RT-PCR primers flanking the HR (Figure 1C). Specificity of the amplicons was assessed by PCR re-amplification analysis using internal RT-PCR primers for each transcript (nested PCR).

### NECC2 is localized to caveolae in PC12 cells

**Figure 2:**
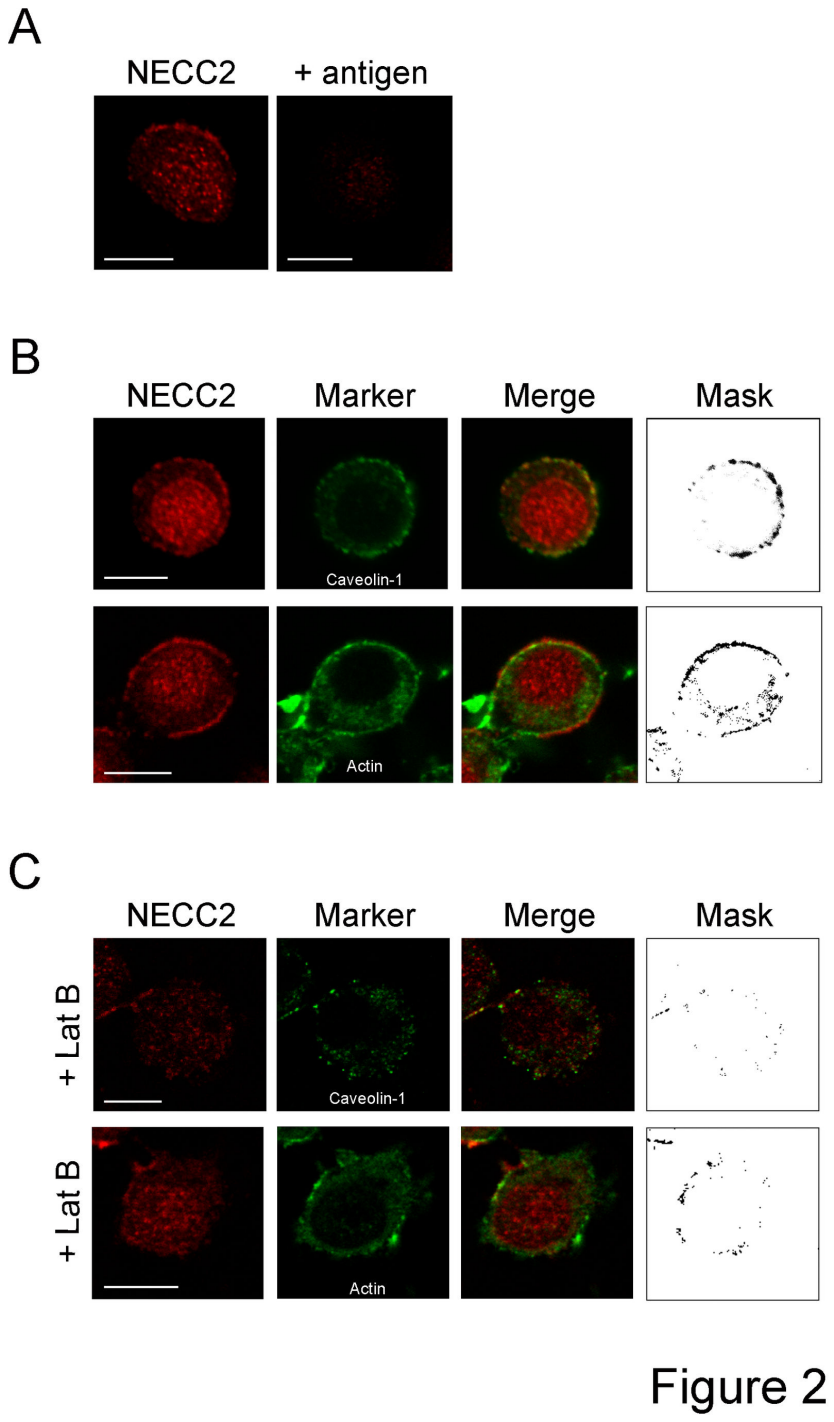
Intracellular distribution of endogenous NECC2 in PC12 cells. *A*. Representative confocal images of a PC12 cell immunolabeled with the anti-NECC2 antiserum. NECC2 distributes throughout the cytoplasm and in close apposition to the cell membrane. Specificity of the signal was tested by preadsorption of the anti-NECC2 antibody with excess of antigen. *B*. PC12 cells were double-stained with antibodies against NECC2 (red) and caveolin-1 or actin (green) (top and bottom panels respectively). Significant overlap between markers at the cell periphery is shown in the binary mask at the rightmost panels. *C*. Prior to double immunostaining with anti-NECC2 and anti-caveolin-1, PC12 cells were treated with 5 µmol/L of LatB for 30 min at 37°C. Scale bars, 10 µm.

Immunostaining of PC12 with a polyclonal antibody raised against the N-terminal region of NECC2 (anti-NECC2) revealed that NECC2 distributed diffusely throughout the cytoplasm and in close apposition to the plasma membrane (Figure 2A). Preadsorption of the anti-NECC2 antibody with its specific antigen (10^-6^ M) resulted in no staining (Figure 2A). The ability of the antibody to recognize NECC2 was confirmed in cMyc-*Necc2*-transfected HEK-293 AD cells, which do not express NECC2 endogenously (Figure S1A). Moreover, anti-NECC2 antibody did not recognize exogenous NECC1 (Figure S1A). The anti-NECC2 antibody also labeled cell nuclei, although this immunosignal was not abolished after preadsorption of the anti-NECC2 serum. A second polyclonal antibody raised against the central region of NECC2 (anti-NECC2-II) confirmed the localization of endogenous NECC2 at the cell surface in PC12 cells (Figure S1B). Similarly, the ability of the anti-NECC2-II antibody to specifically recognize NECC2 was assessed in *Necc2*-transfected HEK-293 AD cells (Figure S1C). The anti-NECC2-II antibody did not recognize exogenous NECC1 in HEK-293 AD cells (Figure S1C).

Given the presence of two overlapping CBDs at the C-terminus of NECC2, we carried out double immunofluorescence in PC12 cells using antibodies against the caveolae marker, caveolin-1, and the anti-NECC2 antibody. Examination by confocal microscopy revealed a high degree of overlap between NECC2 and caveolin-1 at the cell surface (Figure 2B). Immunolabeling of NECC2 with the anti-NECC2-II antibody also showed a high degree of colocalization between NECC2 and caveolin-1 immunosignals (Figure S1B). Moreover, NECC2 immunosignal also colocalized with cortical actin (Figure 2B). Pretreatment of PC12 cells with LatB significantly disrupted both cortical actin and caveolin-1 distribution at the plasma membrane. LatB treatment also prevented NECC2 localization to the cell periphery, leading to a loss of co-localization between NECC2 and caveolin-1 or actin (Figure 2C).

**Figure 3:**
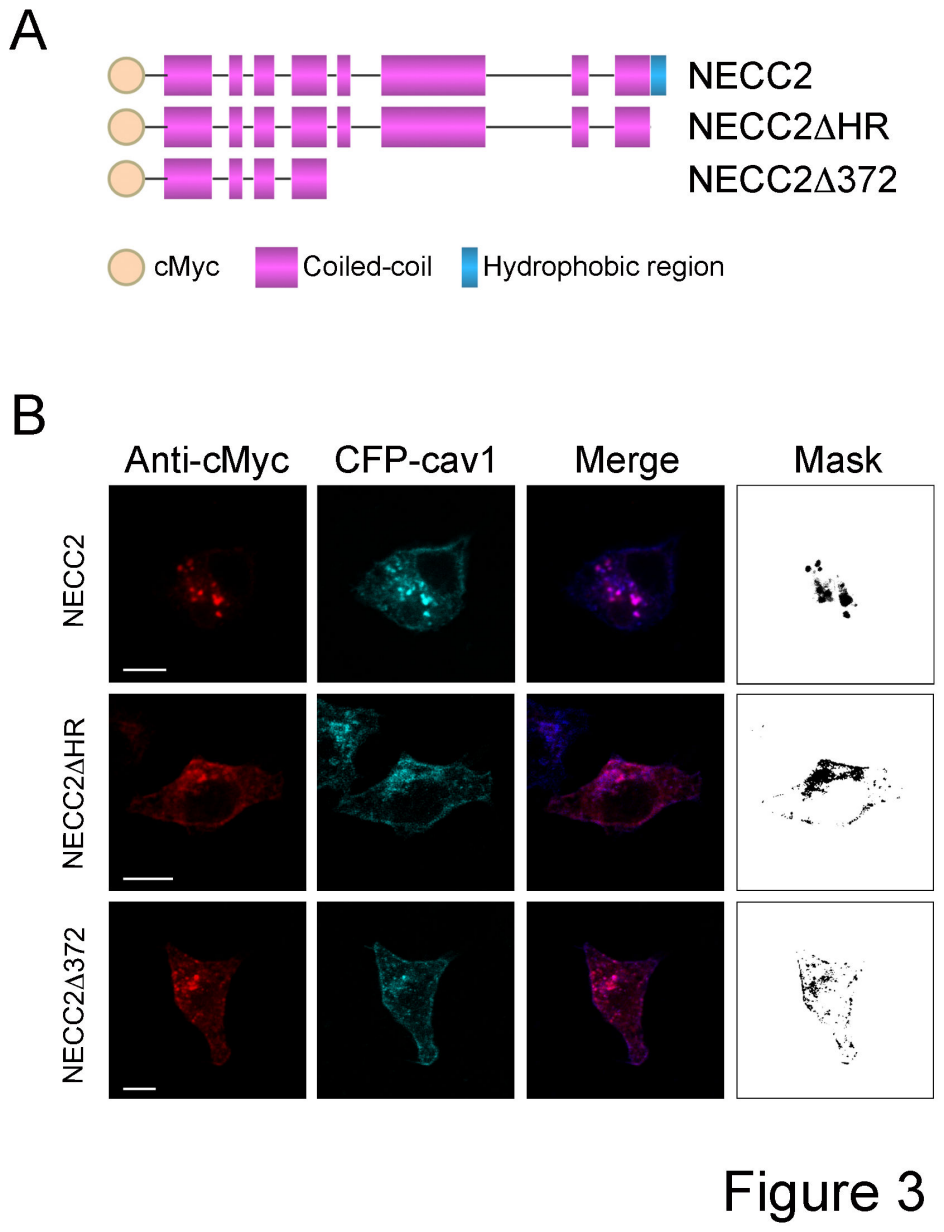
Analysis of the intracellular localization of cMyc-tagged NECC2 variants. *A*. Schematic representation of NECC2 constructs and its truncated variants lacking the hydrophobic region (NECC2ΔHR) or containing the first 4 N-terminal coiled-coil regions (NECC2Δ372). *B*. Confocal images of PC12 cells co-expressing cMyc-NECC2 or its truncated forms and CFP-caveolin-1 (CFP-cav1). Full-length cMyc-NECC2 accumulates in the perinuclear area. NECC2ΔHR and NECC2Δ372 immunosignals accumulate intracellularly and in the proximity of the cell surface. NECC2 immunofluorescence signal strongly overlaps with CFP-caveolin-1 as shown in the binary mask (rightmost panel). Scale bars, 10 µm.

In contrast to that found for the endogenous protein, exogenous cMyc-tagged NECC2 accumulated in the perinuclear region of PC12 cells (Figure S2A). These results are reminiscent of that previously reported for the *Necc2* ortholog, *Necc1*, which forms protein aggregates when transfected in PC12 cells [6]. Likewise, we observed that caveolin-1 accumulated close to the nucleus when exogenously expressed in PC12 cells (Figure S2B), which is in accordance with previous reports in COS-7 and U2OS cells [26,27]. Analysis of PC12 cells co-transfected with cMyc-*Necc2* and CFP-cav1 revealed the colocalization between the two proteins (Figure 3B). We next analyzed the distribution pattern of the deletion mutant of NECC2 lacking the HR domain (NECC2ΔHR) (Figure 3A). As shown in Figure 3B, NECC2ΔHR distributed at the cell surface, wherein it colocalized with caveolin-1. Intracellular accumulation of both proteins was also observed. A shorter truncated form of NECC2 (NECC2Δ372) containing the first 4 N-terminal coiled-coil regions (Figure 3A) also partially overlapped with caveolin-1 at the plasma membrane (Figure 3B). Together, these results suggest that the CBDs are not strictly required for localization of NECC2 to caveolae.

### NECC2 behaves as a caveolae membrane protein

**Figure 4:**
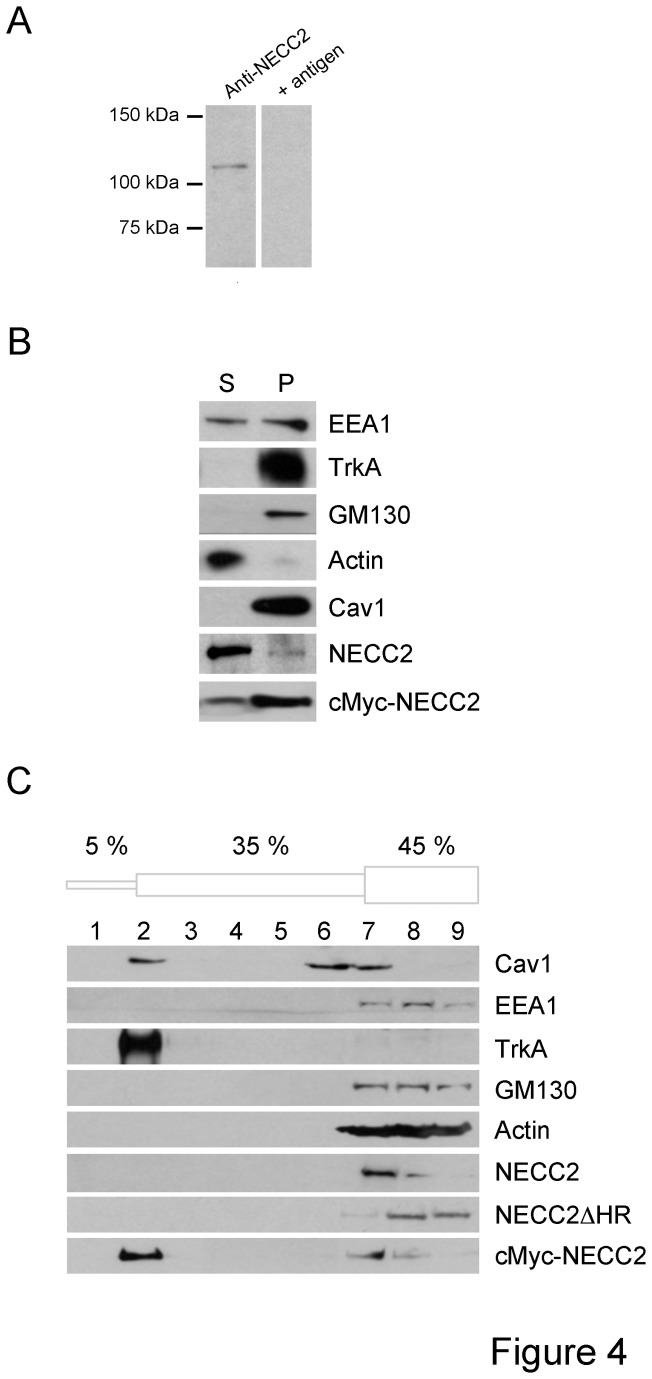
NECC2 associates to caveolae both as an integral membrane protein and a peripheral membrane protein. *A*. Immunoblot analysis of whole cell lysates from PC12 cells with the anti-NECC2 antibody. As control, anti-NECC2 antibody was pre-incubated with an excess of antigen. *B*. Cytosolic (S) and crude membrane (P) fractions from PC12 cells were obtained by subcellular fractionation as described in Methods and subsequent analyzed by immunoblotting. As shown by anti-NECC2 antibody immunolabeling, NECC2 distributes in the cytosol and, to a lesser extent, it also associates with membrane fractions. In contrast, exogenous full-length cMyc-NECC2 distributed to both fractions, with a higher content in the membrane fraction. The distribution of EEA1, TrkA, GM130, actin and caveolin-1 was also analyzed. *C*. Caveolae-enriched membranes from PC12 cells were isolated by using a detergent-free method based in a discontinuous sucrose gradient (5-35-45%). Distribution of endogenous NECC2 and cMyc-tagged NECC2 variants were assayed by immunoblot. Neither NECC2 nor NECC2ΔHR co-migrated with caveolin-1 and TrkA to the buoyant fraction (fraction 2).

Immunoblot analysis of protein extracts from PC12 cells using the anti-NECC2 antibody revealed an immunoreactive band of approximately 110 kDa (Figure 4A), which is close to the expected molecular weight calculated from the cDNA of full-length *Necc2*. Immunodetection was abolished after preadsorption of the antibody with the purified antigen (Figure 4A), as well as in *Necc2*-shRNA (siRNA) transfected cells (see below). The anti-NECC2 antibody strongly immunolabeled exogenous NECC2 in cMyc-NECC2-transfected HEK-293 AD cells, whereas it did not recognize exogenous NECC1 in cMyc-NECC1-transfected cells (Figure S3A). Immunoblotting of protein extracts from PC12 cells using the anti-NECC2-II antibody revealed a similar 110-kDa band, along with two other immunoreactive bands of 140 and 95 kDa (Figure S1D). The NECC2-II antibody recognized exogenous full-length NECC2 but not NECC1 or the truncated form of NECC2 lacking the peptide sequence employed to obtain this antibody, NECC2Δ285 (Figure S3A–C).

To further characterize NECC2, we performed subcellular fractionation studies. Immunoblot analysis of cytosolic and crude membrane fractions from PC12 cells revealed that NECC2 protein is mostly distributed in the cytosol and, to a low extent, associates with membrane fractions (Figure 4B). On the other hand, exogenous cMyc-NECC2 containing the HR domain was distributed in both fractions, with a higher content in membrane fractions (Figure 4B). We next investigated the type of association that NECC2 maintains with caveolae by using a detergent-free method to isolate caveolae membranes based in their “buoyant” properties [15]. As shown in Figure 4C, endogenous NECC2 or NECC2ΔHR did not co-migrate with caveolin-1 and TrkA to “buoyant” fractions. However, the NECC2 isoform containing the HR separated with TrkA and caveolin-1 in the gradient (Figure 4C). These results, together with the microscopic data, suggest that the HR domain is responsible for the integral association of NECC2 to caveolae membranes, although NECC2 may also interact with caveolae components as a peripheral protein.

### NGF regulates NECC2 expression and distribution in PC12 cells

**Figure 5:**
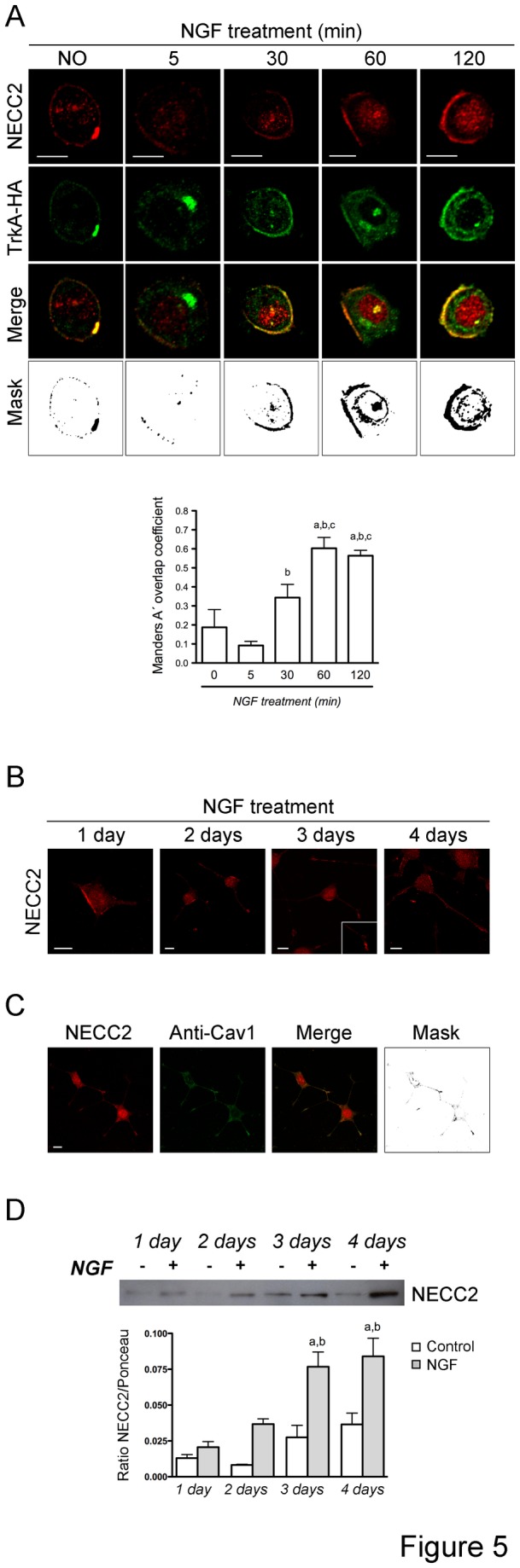
NECC2 expression and distribution are regulated by NGF. *A*. Representative confocal images of HA-*TrkA* transfected PC12 cells under basal conditions or treated with NGF at the indicated time points. After treatment, cells were subjected to double-immunofluorescence using anti-NECC2 and anti-HA antibodies. The colocalization channel was isolated using Imaris 6.4 (Bitplane) and shown alone in the images on the far bottom. Mander’s coefficient A (between NECC2 and TkA) was calculated to quantify the degree of colocalization and represented as the mean ±SEM of at least 5 cells per experimental group. a, *P* < 0.05 *vs*. control; b, *P* < 0.05 *vs*. 5 min; c, *P* < 0.05 *vs*. 30 min (unpaired, 2-tailed t test). *B*. Representative micrographs of PC12 cells immunolabeled with anti-NECC2 during long-term (1-4 days) stimulation with NGF. NECC2 staining localizes beneath the plasma membrane as well as in puncta/vesicular-like structures in growing neurites and tips. *C*. Double-immunostaining of NGF-differentiated PC12 cells with anti-NECC2 and anti-caveolin-1 sera. Scale bars, 10 µm. *D*. Protein extracts from NGF-stimulated PC12 cells during differentiation (1-4 days) were analyzed by immunoblotting using the anti-NECC2 antibody. Quantitative data were represented as ratio of NECC2 vs. Ponceau. The data represent the mean (± SEM) of three independent experiments. a, *P* < 0.05 *vs*. corresponding control; b, *P* < 0.05 *vs*. 1- or 2-days treated cells (one-way ANOVA followed by *Newman–Keuls* test).

The intracellular distribution of NECC2 during NGF-induced PC12 cell differentiation was also investigated. Short-term treatment of PC12 cells with NGF (0-120 min) increased the association of NECC2 to the cell surface (Figure 5A). Specifically, the percentage of cells with increased surface-associated NECC2 immunoreactivity augmented from 50 to 66.0% after a 1-h exposure to NGF. Immunolabeling of NECC2 and exogenous HA-TrkA demonstrated that these proteins colocalized at the cell surface of PC12 cells under basal conditions (MC between NECC2 and TrkA was 0.187). Colocalization between NECC2 and TrkA was transiently decreased by 5 min and subsequently increased at later points after short-term stimulation with NGF (MC = 0.092, 0.343, 0.603, and 0.564 at 5, 30, 60, and 120 min of NGF stimulation, respectively). NECC2 immunoreactivity at the cell surface was maintained in PC12 cells after long-term exposure to NGF (1-4 days) (Figure 5B). Along with this distribution, NECC2 immunosignal also localized to puncta/vesicular-like structures in growing neurites and tips (Figure 5B), wherein it colocalized with caveolin-1 (Figure 5C).

In line with the microscopic data, immunoblot analysis revealed that NECC2 protein levels increased progressively during NGF-induced differentiation of PC12 cells (Figure 5D).

### NECC2 regulates NGF-mediated TrkA signaling

**Figure 6:**
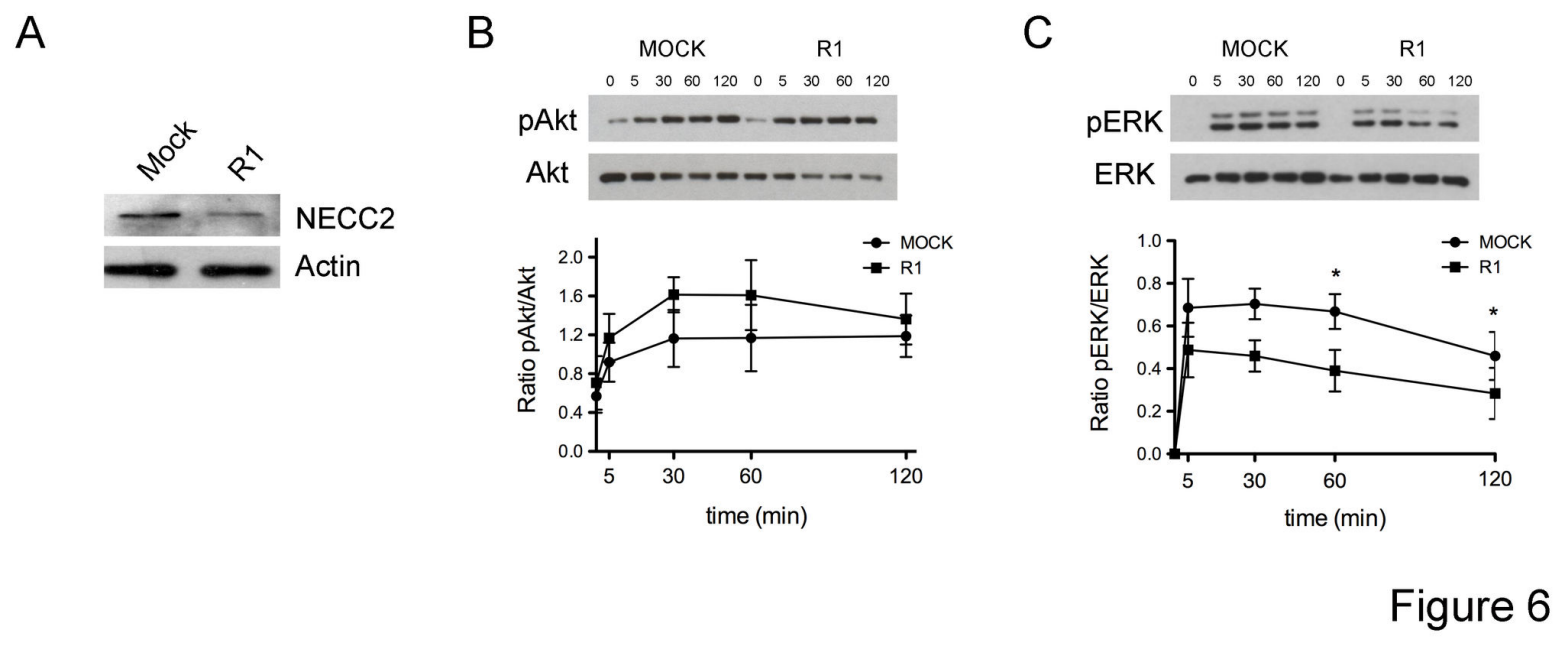
Reduction of endogenous NECC2 by RNA interference impairs NGF-mediated TrkA signaling. *A*. PC12 cells were stably transfected with pEGFP-shRNA or *Necc2*-shRNA plasmid (siRNA) and whole protein extracts were analyzed for immunoblotting using anti-NECC2 antibody and anti-actin antibody. *B* and C. PC12 cells overexpressing pEGFP-RNAi (MOCK) or *Necc2*-shRNA (R1) were grown to 90% confluence and exposed for 4 h to serum-low differentiation media before NGF stimulation for the indicated time points. Cell lysates were subjected to immunoblot with Akt and phospho-Akt (pAkt) antibodies (*B*) or with ERK and phospho-ERK (pERK) antibodies (*C*). Quantitative data were represented as ratio of pAkt *vs*. Akt or pERK vs. ERK, respectively. The data represent the means (± SEM) of three independent experiments. *P* < 0.05 *vs*. corresponding control (unpaired, 2-tailed t test).

Given the NGF-dependent colocalization of NECC2 and TrkA, we examined the effects of *Necc2* silencing and overexpression on the phosphorylation rate of the main TrkA-activated effectors, ERK1/2 and Akt, in NGF-treated cells. To reduce NECC2 expression, PC12 cells were stably transfected with an expression vector encoding a shRNA for *Necc2* (siRNA) (Figure 6A). Down-regulation of NECC2 expression had no effect on cell viability as determined by the MTT assay (0,1693 ± 0.0546 in mock-transfected cells vs. 0,1582 ± 0.0197 in *Necc2* siRNA-expressing cells). Decreased concentrations of NECC2 did not affect Akt phosphorylation at any time point analyzed (Figure 6B). In contrast, phosphorylation of ERK1/2 was decreased in cells with reduced expression of NECC2. Specifically, ERK phosphorylation ratio was numerically lower in silenced cells than in mock-transfected cells at all the time points tested, although differences between the two groups reached statistical significance after 60 and 120 min of exposure to NGF.

**Figure 7:**
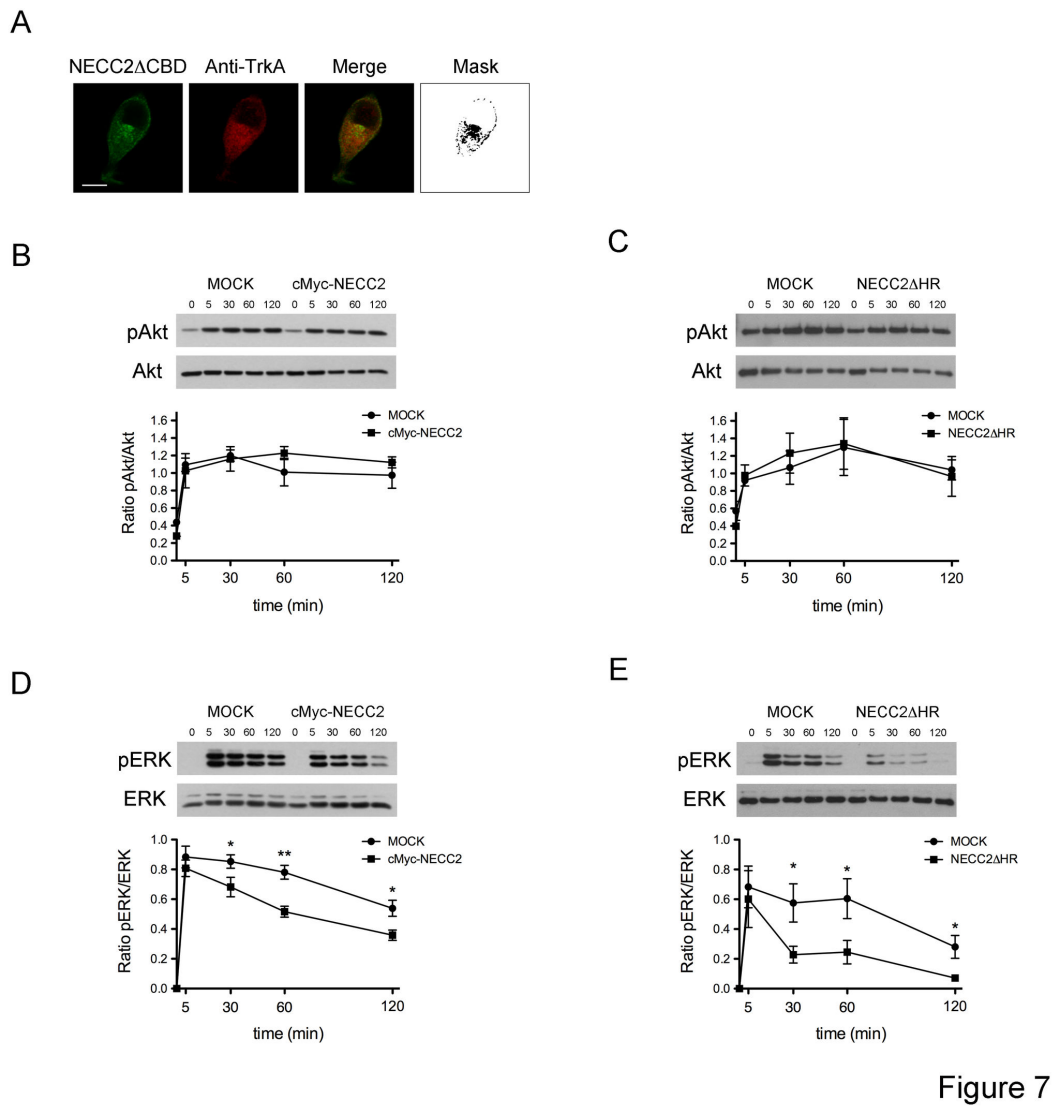
Overexpression of NECC2 inhibits NGF-mediated TrkA signaling pathway. *A*. Representative confocal images of PC12 cells transfected with cMyc-*Necc2*ΔHR and double-stained with anti-cMyc and anti-TrkA antibodies. Immunofluorescent signals significantly overlap at the cell periphery and intracellularly as shown in the binary mask (right panels). Scale bars, 10 µm. *B*, *C*, *D* and *E*. PC12 cells transiently transfected with full-length cMyc-*Necc2*, cMyc-*Necc2*ΔHR, or the empty vector (mock) were grown to 90% confluence and exposed for 4 h to serum-low differentiation media before NGF stimulation for the indicated time points. Whole cell protein extracts were then subjected to immunoblot with Akt and phospho-Akt (pAkt) antibodies (*B* and *C*) or with ERK and phosphor-ERK (pERK) antibodies (*D* and *E*). Quantitative data were represented as ratio of pAkt *vs*. Akt or pERK vs. ERK, respectively. The data represent the means (± SEM) of three independent experiments. *P* < 0.05 *vs*. corresponding control (unpaired, 2-tailed t test).

Finally, we examined the effect of NECC2 overexpression on the phosphorylation of Akt and ERK1/2. To this end, PC12 cells were transfected with the NECC2 isoform lacking the HR domain, which colocalizes with TrkA, both at the cell surface and intracellularly (Figure 7A), or with the long NECC2 isoform. Overexpression of NECC2 had no effect on cell viability (data not shown). Similar to that found in the silencing experiments, increased expression of NECC2 or NECC2ΔHR did not modify NGF-induced Akt phosphorylation (Figure 7B and 7C, respectively). Up-regulation of NECC2 expression significantly decreased NGF-activated ERK1/2 phosphorylation at 30, 60, and 120 min of exposure to the neurotrophin (Figure 7D and 7E).

## Discussion

Caveolae are cholesterol- and sphingolipid-rich membrane microdomains, which, among other key functions, constitute specialized signaling platforms that allow physical localization and direct interaction of multiple types of receptors with their intracellular mediators [28]. These specialized domains cluster a variety of specific proteins, including the structural proteins caveolins and cavins, along with several cytoskeletal proteins and scaffolding factors which are essential for caveolae formation, maintenance, and functional organization [12,13,29–31].

Fluorescence microscopy using two different anti-NECC2 antibodies revealed that NECC2 co-localized with caveolin-1 at the cell surface of PC12 cells. NECC2 immunosignal was also closely associated with cortical actin, which has been shown to regulate the dynamics and localization of caveolin-1 in caveolae [32]. Accordingly, disruption of actin filaments by LatB treatment altered NECC2 distribution and decreased significantly the co-localization between NECC2 and both caveolin-1 and actin. In all, these observations indicated that NECC2 locates to caveolae in PC12 cells. These results were in line with our in silico analysis showing the presence of two CBD domains as well as four CRAC motifs in NECC2 sequence, which have been proposed to promote the incorporation of proteins into cholesterol-rich domains [21,33]. Nevertheless, recent data indicates that CBDs are not enriched among caveolin-binding proteins and, more importantly, that the interaction between CBDs and the scaffolding domain in caveolin-1 does not likely mediate caveolae-associated signaling [34]. In fact, our microscopy studies using truncated NECC2 variants support the notion that neither the CBDs nor the CRAC domains are required for the localization of NECC2 to caveolae. Alternatively, NECC2 targeting to caveolae might occur via the interaction of its coiled-coil domains with caveolae-resident proteins. In this regard, a role for coiled-coil domains in intracellular targeting has been reported for the long coiled-coil proteins associated to the Golgi apparatus, golgins [11].

Subcellular fractionation studies showed that endogenous NECC2 distributed mainly in the cytosol and, to a lesser extent, in membrane fractions. Detergent-free extraction of caveolae-enriched membranes and sucrose gradient fractionation analysis confirmed that both the endogenous protein and the NECC2 variant lacking the HR were located in non-buoyant fractions. However, full-length NECC2 containing the HR migrated to caveolae-enriched membrane fractions. These observations, which are consistent with the expression of several NECC2 variants in PC12 cells, suggest that NECC2 associates to caveolae membranes as an integral or a peripheral membrane protein depending on the presence or absence of the HR domain, respectively. In line with these findings, the family of long coiled-coil proteins ERC comprises two distinct members generated by alternative splicing, ERC1a and ERC1b, that differ in both their expression pattern and intracellular distribution [35]. Thus, whereas ERC1a is a cytosolic protein, ERC1b is both a cytosolic protein and an insoluble component of the active zone at the synapse [35]. Interestingly, golgins undergo disassociation/association cycles between the cytosol and Golgi membranes [36]. Likewise, it has been shown that soluble cavin proteins peripherally attach to caveolae once they reach the cell surface [29]. In this scenario, the NECC2 isoform lacking the HR might cycle between a soluble, cytosolic pool and the caveolae membrane, likely via its interaction with a caveolae-associated protein.

Neurotrophic factor receptors have been found associated with lipid rafts in several cell types [16,37–40]. It has been shown that TrkA receptor accumulation in lipid rafts facilitates receptor dimerization and transphosphorylation [16,41]. Herein, we observed that NECC2 colocalized at the cell surface with the TrkA receptor, which has been located to caveolae in PC12 cells [15] (our unpublished observations). However, the extent of colocalization between NECC2 and TrkA varied in a time-dependent manner following NGF stimulation. Thus, this colocalization transiently decreased by 5 minutes of NGF stimulation, likely due to NGF-induced TrkA internalization, and increased subsequently to reach a maximum after 60 minutes of exposure to NGF. In line with this, it has been shown that, following NGF-evoked internalization, TrkA receptors are recycled back to the plasma membrane [42]. We observed that both the percentage of PC12 cells showing accumulated NECC2 at the plasma membrane and the amount of NECC2 immunosignal at the cell surface increased upon NGF exposure, suggesting that this factor induces the recruitment of NECC2 to cell surface microdomains. Similar results have been reported for the adaptor protein that is responsible of TrkA localization to lipid rafts, c-Cbl associated protein (CAP), which also moves to the cell surface following short-term NGF stimulation of PC12 cells [41].

Our microscopic and biochemical findings suggested that NECC2 might regulate TrkA-mediated NGF signaling in caveolae. In support of this proposal are our functional studies showing that decreasing NECC2 content by shRNA-mediated (siRNA) silencing altered NGF-induced phosphorylation of the TrkA downstream effector ERK1/2. Remarkably, NECC2 silencing had no effect on NGF-induced activation of Akt, which is also mediated via NGF binding to TrkA [43,44]. Overexpression of NECC2 mimicked the effects evoked by NECC2 silencing on both signaling routes. When viewed together, these data suggest that appropriate expression levels of NECC2 are required for proper TrkA activity and strongly support a role for this protein in the regulation of caveolae function. In addition, they suggest that, when overexpressed, NECC2 may display a dominant-negative effect, as it has been previously reported for other long coiled-coil proteins [45]. Notably, it has been shown that overexpression of caveolin-1 functions as a dominant negative [26] and impairs NGF-induced differentiation of PC12 cells [46].

Trafficking, sorting, and ubiquitination of ligand-activated TrkA receptors regulate the magnitude and duration of the intracellular response evoked by NGF [42]. It is well established that TrkA activation triggers a transient, short-term response (5 min) that involves the activation of both the PI3K/Akt and the ERK/MAPK pathways [44]. Once in endosomes, TrkA induces the selective activation of ERKs via the small GTPase Rap1 and B-Raf [44,47]. In our study, we observed that changes in NECC2 expression levels impaired sustained ERK1/2 phosphorylation in response to NGF treatment, without affecting NGF-induced Akt activation. Similar results have been depicted for several TrkA-related signaling adapter proteins located to lipid rafts, such as FRS2 and ARMS/kidins220 [48–51]. In particular, both FRS2 and ARMS/kidins220 are involved in the formation of the signalling complex Crk-C3G, which is responsible of targeting NGF signaling to Rap1-ERKs [48–50]. Crk possesses SH3 and SH2 domains, which are essential for its interaction with ARMS/kidins220 and FRS2 [51,52]. Interestingly, NECC2 possesses a proline-rich motif (PXXP), which is considered a target domain for proteins containing SH3-type motifs [25]. Given this and the structural features conferred by the coiled-coil domains [7], together with our functional data, it is tempting to speculate that NECC2 might act as a scaffolding factor facilitating the interaction of the signaling molecules responsible of NGF-dependent persistent ERK activation. Alternatively, NECC2 could play a role in TrkA compartmentalization and/or trafficking towards the endosomal system by acting as a tethering factor. In line with this proposal, golgins have been shown to facilitate sorting and membrane trafficking across the Golgi apparatus by regulating membrane-membrane and membrane-cytoskeleton tethering events [11,53,54]. Likewise, the long coiled-coil proteins liprins and ERC2/CAST, which are located to the cytomatrix at the active zone of synapses, act as regulators of membrane fusion events and stabilize these membrane microdomains [9,10,55,56]. Interestingly, NECC2 distributed in neurites and growth cones in differentiated PC12 cells, wherein it largely colocalized with caveolin-1. It has been reported that caveolin-1 recruits synaptic components and regulates signal transduction of a variety of neurotransmitter and neurotrophic receptors in the CNS [57], thus suggesting its participation in synapse maturation and/or remodelation [58–60]. In this scenario, a general role for NECC2 as organizer of membrane microdomains specialized in signal transduction seems plausible.

In sum, our data are the first to demonstrate the presence of a long coiled-coil protein, NECC2, in caveolae, and to identify a potential regulatory role for this protein in the control of the NGF-TrkA system in PC12 cells.

## Supporting Information

Figure S1 **Associated with Figure 2.** Analysis of the specificity of anti-NECC2 and anti-NECC2-II antibodies by immunostainig. *A*. HEK293 AD cells were transfected with full-length cMyc-*Necc2* or cMyc-*Necc1* and doubled-stained with anti-cMyc (green) and anti-NECC2 antibodies (red). As shown in the binary mask (right panels), the anti-NECC2 antibody recognized exogenous NECC2, but not NECC1. Scale bars, 10 µm *B*. PC12 cells were immunolabeled with anti-NECC2-II (red) and anti-caveolin1 (green) antibodies. NECC2 distributes at the plasma membrane (left panel). Significant colocalization between NECC2 and caveolin-1 (green) is shown in the binary mask at the rightmost panels. *C*. cMyc-*Necc2*- or cMyc-*Necc1*-transfected HEK293 AD cells were immunolabeled with anti-cMyc (green) and anti-NECC2-II (red) antibodies. Anti-NECC2-II antibody recognized exogenous NECC2 but not NECC1. *D*. Immunoblot analysis of PC12 cell lysates using the anti-NECC-II antibody revealed a 110-kDa immunoreactive band along with two other bands of 140 and 95 kDa.
(TIF)

Figure S2 **Associated with Figure 3.** Intracellular distribution of exogenous NECC2 and Caveolin1. *A*. PC12 were transfected with cMyc-*Necc2*, GFP-*Necc2*, or untagged *Necc2* and stained with anti-cMyc (leftmost panel) or anti-NECC2 antibodies (rightmost panel). Regardless of the reporter sequence used, NECC2 exhibited a juxtanuclear distribution in transfected PC12 cells. *B*. Representative confocal images of PC12 cells transfected with CFP-*Caveolin-1* (GFP-cav1). CFP-cav1 located to the plasma membrane and also accumulated close to the nucleus, as has been previously reported for other cell types [26,27]. Scale bars, 10 µm.
(TIF)

Figure S3 **Associated with Figure 4.** Analysis of the specificity of anti-NECC2 and anti-NECC2-II antibodies by immunoblotting. *A*. HEK293 AD cells were transfected with cMyc-*Necc2* or cMyc-*Necc1* and analyzed by immunoblotting using anti-cMyc, anti-NECC2, and anti-NECC2-II antibodies. Non-transfected HEK293 AD cells were used as controls. Exogenous NECC2 protein, but not NECC1, was detected with the anti-NECC2 and the anti-NECC-II antibodies. *B*. Immunoblot analysis of whole cell lysates from cMyc-*Necc1*- or cMyc-*Necc2*Δ285- transfected HEK293 AD cells. Anti-NECC-II antibody did not recognize cMyc-*Necc2*Δ285, the truncated form of NECC2 lacking the peptide sequence employed to obtain the anti-NECC2-II antibody. *C*. HEK293 AD cells were transfected with cMyc-*Necc2*Δ285 and double-stained with anti-cMyc (green) and anti-NECC2 antibodies (red). As shown in the images, anti-NECC2-II antibody did not recognize the truncated form, cMyc-NECC2Δ285. Scale bars, 10 µm.
(TIF)
