# Correction: The Long Coiled-Coil Protein NECC2 Is Associated to Caveolae and MODULATES NGF/TrkA Signaling IN PC12 CELLS

**DOI:** 10.1371/annotation/55c4c5bd-99e8-435a-94f4-7e151043bb51

**Published:** 2013-09-12

**Authors:** Alberto Díaz-Ruiz, Yoana Rabanal-Ruiz, Andrés Trávez, Francisco Gracia-Navarro, David Cruz-García, Maité Montero-Hadjadje, Youssef Anouar, Stéphane Gasman, Nicolas Vitale, Rafael Vázquez-Martínez, María M. Malagón

There were errors in the article title.

The correct version of the title is: The Long Coiled-Coil Protein NECC2 Is Associated to Caveolae and Modulates NGF/TrkA Signaling in PC12 Cells

The correct version of the citation is: Díaz-Ruiz A, Rabanal-Ruiz Y, Trávez A, Gracia-Navarro F, Cruz-García D, et al. (2013) The Long Coiled-Coil Protein NECC2 Is Associated to Caveolae and Modulates NGF/TrkA Signaling in PC12 Cells. PLoS ONE 8(9): e73668. doi:10.1371/journal.pone.0073668 

