# Supplementary figures and images for: The Long Coiled-Coil Protein NECC2 Is Associated to Caveolae and MODULATES NGF/TrkA Signaling IN PC12 CELLS
Source: PLoS One. 2013 Sep 6;8(9):e73668. doi: 10.1371/journal.pone.0073668 (PMC3765260; doi:10.1371/journal.pone.0073668)

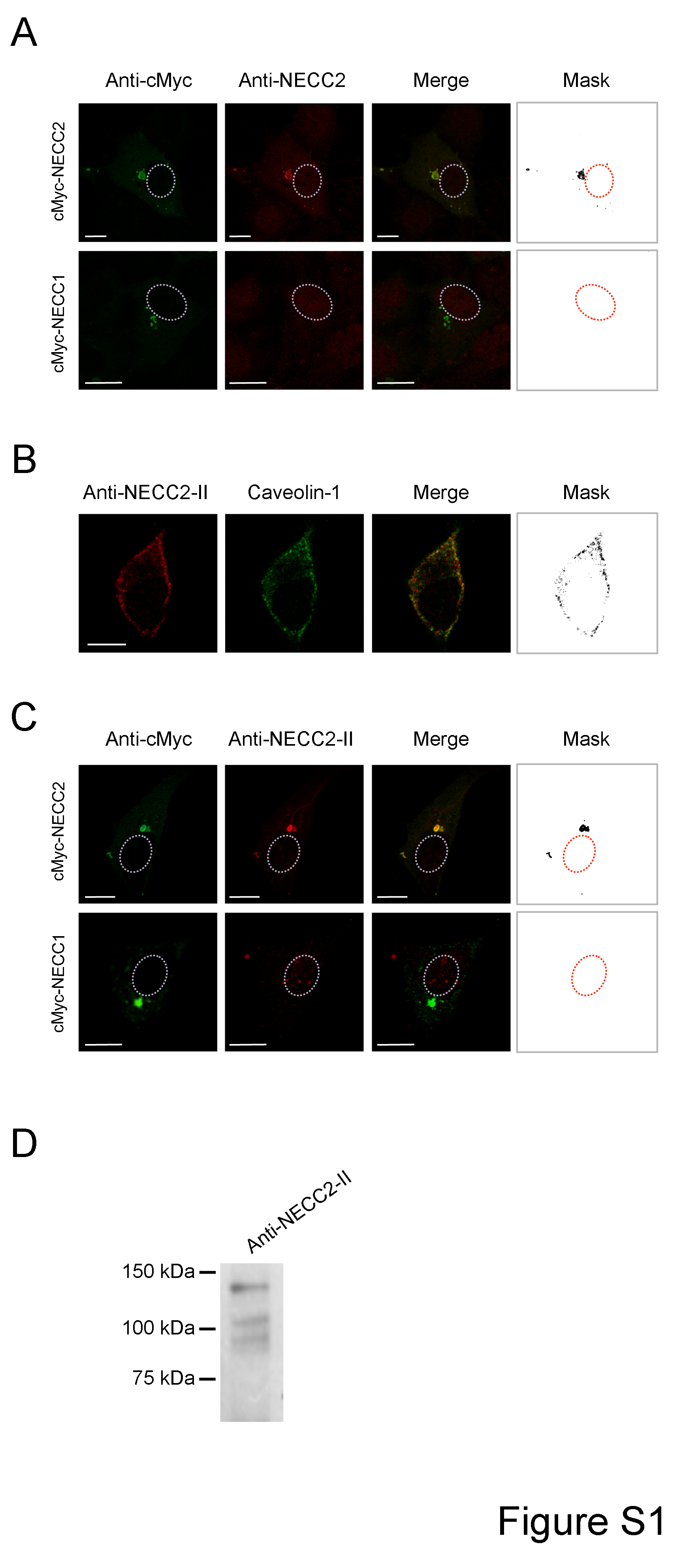

Supplement: Figure S1 — Associated with Figure 2. Analysis of the specificity of anti-NECC2 and anti-NECC2-II antibodies by immunostainig. A. HEK293 AD cells were transfected with full-length cMyc-Necc2 or cMyc-Necc1 and doubled-stained with anti-cMyc (green) and anti-NECC2 antibodies (red). As shown in the binary mask (right panels), the anti-NECC2 antibody recognized exogenous NECC2, but not NECC1. Scale bars, 10 µm B. PC12 cells were immunolabeled with anti-NECC2-II (red) and anti-caveolin1 (green) antibodies. NECC2 distributes at the plasma membrane (left panel). Significant colocalization between NECC2 and caveolin-1 (green) is shown in the binary mask at the rightmost panels. C. cMyc-Necc2- or cMyc-Necc1-transfected HEK293 AD cells were immunolabeled with anti-cMyc (green) and anti-NECC2-II (red) antibodies. Anti-NECC2-II antibody recognized exogenous NECC2 but not NECC1. D. Immunoblot analysis of PC12 cell lysates using the anti-NECC-II antibody revealed a 110-kDa immunoreactive band along with two other bands of 140 and 95 kDa. (TIF) [file pone.0073668.s001.tif]

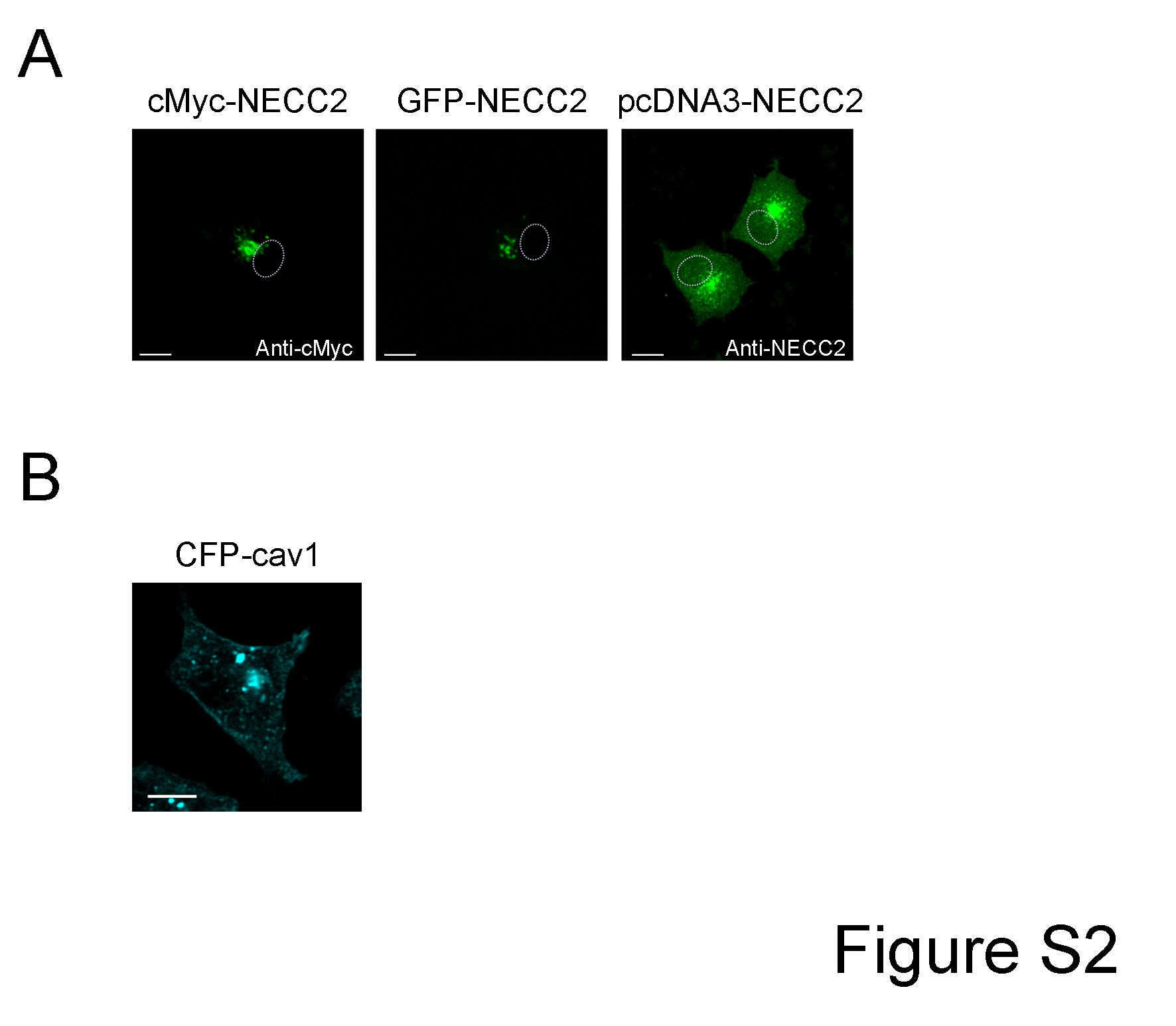

Supplement: Figure S2 — Associated with Figure 3. Intracellular distribution of exogenous NECC2 and Caveolin1. A. PC12 were transfected with cMyc-Necc2, GFP-Necc2, or untagged Necc2 and stained with anti-cMyc (leftmost panel) or anti-NECC2 antibodies (rightmost panel). Regardless of the reporter sequence used, NECC2 exhibited a juxtanuclear distribution in transfected PC12 cells. B. Representative confocal images of PC12 cells transfected with CFP-Caveolin-1 (GFP-cav1). CFP-cav1 located to the plasma membrane and also accumulated close to the nucleus, as has been previously reported for other cell types [26,27]. Scale bars, 10 µm. (TIF) [file pone.0073668.s002.tif]

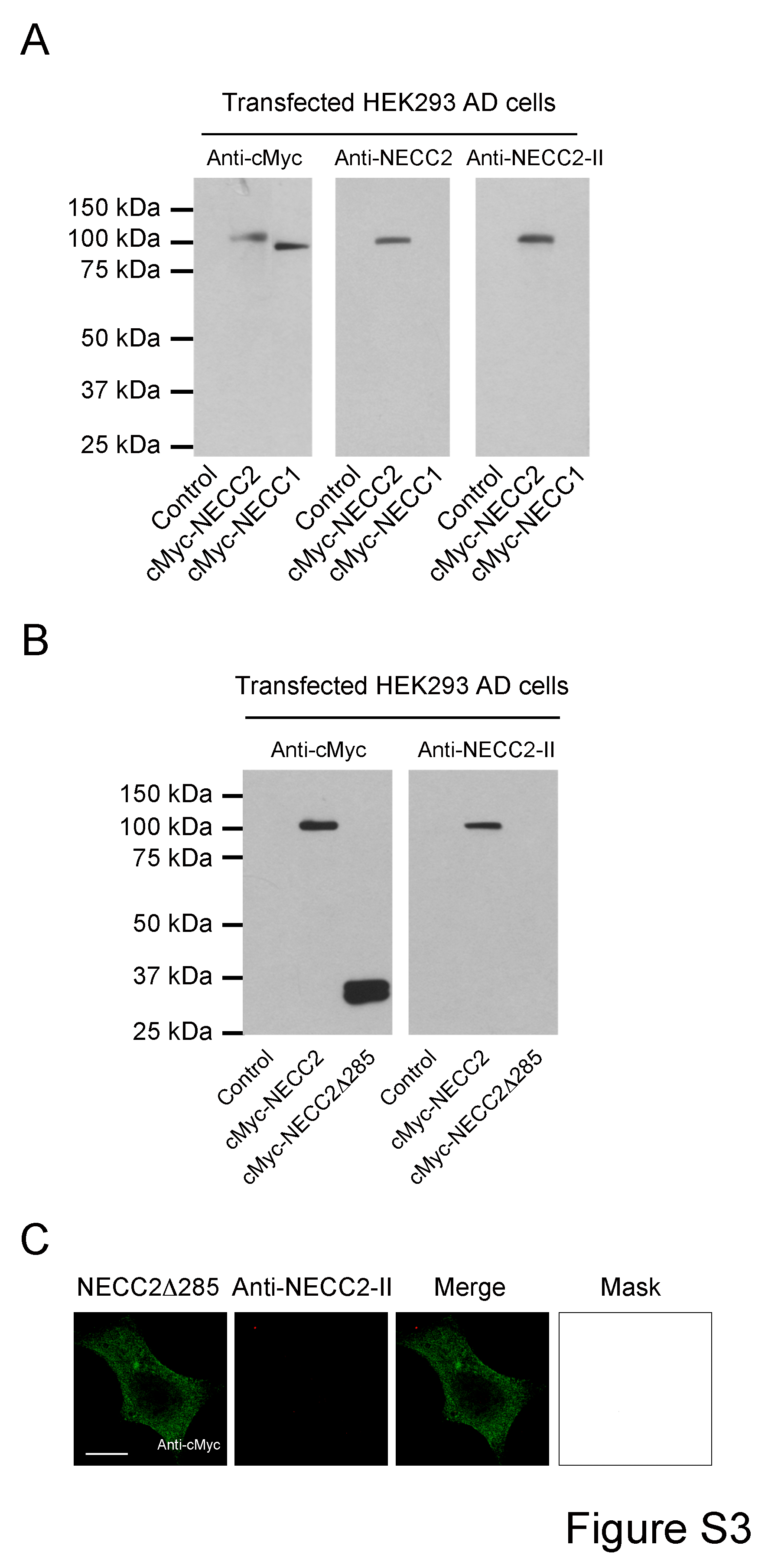

Supplement: Figure S3 — Associated with Figure 4. Analysis of the specificity of anti-NECC2 and anti-NECC2-II antibodies by immunoblotting. A. HEK293 AD cells were transfected with cMyc-Necc2 or cMyc-Necc1 and analyzed by immunoblotting using anti-cMyc, anti-NECC2, and anti-NECC2-II antibodies. Non-transfected HEK293 AD cells were used as controls. Exogenous NECC2 protein, but not NECC1, was detected with the anti-NECC2 and the anti-NECC-II antibodies. B. Immunoblot analysis of whole cell lysates from cMyc-Necc1- or cMyc-Necc2Δ285- transfected HEK293 AD cells. Anti-NECC-II antibody did not recognize cMyc-Necc2Δ285, the truncated form of NECC2 lacking the peptide sequence employed to obtain the anti-NECC2-II antibody. C. HEK293 AD cells were transfected with cMyc-Necc2Δ285 and double-stained with anti-cMyc (green) and anti-NECC2 antibodies (red). As shown in the images, anti-NECC2-II antibody did not recognize the truncated form, cMyc-NECC2Δ285. Scale bars, 10 µm. (TIF) [file pone.0073668.s003.tif]
